# Traditional and modern educational resource use in Emergency Medicine registrars (trainees) and specialists in South Africa

**DOI:** 10.1016/j.afjem.2026.100985

**Published:** 2026-06-22

**Authors:** Almien Smit, Vidya Lalloo

**Affiliations:** a Emergency medicine specialist, Tembisa Provincial Tertiary Hospital, Gauteng Department Health, Division of Emergency Medicine, University of Pretoria, Bophelo Rd, Pretoria, South Africa; b Emergency medicine specialist, Kalafong Provincial Tertiary hospital, Division of Emergency Medicine, University of Pretoria, Bophelo Rd, Pretoria, South Africa; c Division of Emergency medicine, University of Pretoria, South Africa

**Keywords:** FOAM, Social media, Modern resources, Traditional resources, EM education, Health education, Medical education, Emergency care

## Abstract

**Background:**

The rise of Free Open Access Medical Education (FOAM) has transformed how Emergency Medicine (EM) practitioners access educational content. While widely adopted globally, its use in South Africa has not been well studied. This study aimed to assess the use of modern (FOAM/social media) and traditional (textbooks/journals) educational resources among EM faculty in Gauteng, South Africa.

**Methods:**

A descriptive, cross-sectional survey was conducted among EM specialists and registrars (trainees/ residents) affiliated with the University of the Witwatersrand and the University of Pretoria from October 2023 to December 2024. A self-administered online questionnaire was used to collect data on resource preferences, frequency of use, perceived quality, reasons for use, and barriers to access. Descriptive statistics were used to analyse the data.

**Results:**

Fifty-five responses were received (76% registrars, 24% consultants). FOAM use was high, with 100% of respondents using blogs, 98% using podcasts, and 91% using social media for EM education. Textbooks and journals remained widely used (89%), particularly for in-depth learning. EMCrit and Life in the Fast Lane (LITFL) were the most frequently accessed FOAM resources. The social media platform X (formerly Twitter) was commonly used on a daily basis for educational purposes. In contrast, restricted-access platforms like Challenger and ENLIGHTENme were used infrequently. The most cited reason for FOAM use was EM education (93%), while point-of-care learning was less common (69%). Notably, 22% of respondents overall reported never checking the references of FOAM content. All respondents reported adequate internet access, with no barriers related to connectivity.

**Conclusion:**

Modern educational resources such as FOAM and social media are widely used among EM faculty, with traditional resources still valued for foundational knowledge. Respondents preferred modern resources for their accessibility and relevance, and no major barriers to FOAM use were identified. These findings support the integration of high-quality FOAM content into EM education, alongside strategies to strengthen critical appraisal skills.

## African relevance

• This study explores the educational resource preferences of emergency medicine faculty in South Africa, a middle-income country, with growing reliance on digital tools for clinical training.

• This study provides context-specific insights into Free Open Access Medical education (FOAM) use within an urban, middle-income African setting, highlighting potential implications for similar training environments while contrasting it with trends in both high-income and other low- and middle-income countries.

• Our findings demonstrate widespread adoption of FOAM within a hybrid learning model, highlighting opportunities to integrate these resources into formal curricula while strengthening critical appraisal skills.

## Introduction

The past decade has seen the rapid rise of Free Open Access Medical Education (FOAM), a movement that emerged alongside the growth of social media [1]. The term “FOAM” was coined by the Emergency Medicine (EM) and critical care community in 2012 [1]. It refers to a collection of freely accessible, interactive online educational resources aimed at both undergraduate and postgraduate medical learners, as well as healthcare professionals. These resources span various digital platforms, including blogs, podcasts, videos, and other web-based media [2].

In addition to dedicated websites, social media platforms contribute significantly to the FOAM ecosystem. Globally, Facebook has approximately 3.07 billion monthly active users, while X (formerly Twitter) has an estimated 550 million monthly active users globally, although figures vary depending on reporting methodology [3]. Studies suggest that between 45% and 96% of healthcare professionals, across all stages of training, have Facebook accounts [4]. These platforms enable informal, peer-driven learning and discussion.

FOAM resources are accessible at the bedside via mobile devices, are often engaging and interactive, and offer flexibility—for example, podcasts can be listened to during commutes, effectively extending study time. This makes FOAM particularly valuable for EM training in low- and middle-income countries [2].

However, FOAM usage is concentrated in a handful of high-income countries—namely the United States, Australia, Canada, and the United Kingdom—who also dominate content creation [2]. In contrast, uptake remains lower in parts of South America, central Africa, and Asia, where economic and language barriers limit access [2]. Notably, South Africa, despite being a middle-income country, recorded 195,070 FOAM sessions in 2016, ranking 12th globally [2]. In this context, a “session” refers to a single instance of a user interacting with a FOAM website, typically defined by a series of page views within a specified time period before inactivity resets the session [4].

There remains a paucity of data on FOAM usage in South Africa. To date, only one study—focused on a limited population in Cape Town—has been published [5]. Further research is needed to explore the extent and nature of FOAM engagement across the country. Understanding user preferences across the EM faculty will inform the development of tailored educational strategies. In addition, identifying barriers to FOAM use is essential to facilitate its effective integration into training programmes.

Gauteng is home to two out of the six medical faculties in South Africa, featuring over six academic hospitals. In South Africa, EM training follows a registrar-based postgraduate model, where trainees are employed within a university-linked training programme and rotate through multiple public sector hospitals within a defined academic complex. In the South African context, a registrar refers to a doctor enrolled in a specialist postgraduate training programme (equivalent to a resident in other settings).

In Gauteng, these programmes are based at large tertiary and regional hospitals serving high-volume, resource-variable populations, providing a diverse clinical training platform. This provides a strong population base for further evaluation of resource use in EM, comparing modern and traditional practices in South Africa.

The aim of this study was to evaluate the use of modern (FOAM and social media) and traditional (textbooks and journals) educational resources among emergency medicine registrars and specialists in Gauteng. Specifically, we sought to describe patterns of use, perceptions of quality and currency, and the factors influencing resource preferences.

## Methods

This was a cross-sectional descriptive study. The study was conducted among EM registrars (postgraduate trainees) and specialists affiliated with the University of Pretoria (UP) and the University of the Witwatersrand (Wits). The study employed a census approach, inviting all eligible EM specialists and registrars at both institutions to participate. The survey link was sent to the academic program heads of both universities, who were asked to distribute it to all the registrars and specialists in their program in accordance with the POPI act. The academic program heads were also contacted periodically to request that a reminder be sent to all the participants in their program. Participation was entirely voluntary and it was made clear to participants that they could withdraw at any time.

All data was collected by the investigator using an online FormsApp platform. Data collection took place from October 2023 to December 2024. Because the study aimed to include the full accessible population, the survey period was extended to allow additional reminders and improve the response rate.

The questionnaire used in the survey was adapted from the one developed by Kleynhans et al. [5] and updated to incorporate modern educational resources. Its use allowed for contextual relevance and facilitated comparison with existing local data. Two additional questions were included based on the literature review to capture key aspects of digital learning behaviour not fully addressed in the original instrument, specifically practices related to verification of FOAM content (e.g. checking references) and the purposes of resource use (e.g. education, procedural learning, and point-of-care decision-making). These additions allowed for comparison with existing literature and ensured alignment with the study objectives. Questions related to EMS and nursing were removed, as these groups were not part of the study population. The questionnaire is available as Appendix A.

An information page accompanied the online survey. Consent was implied by voluntary completion and submission of the questionnaire. No personally identifying information was collected, ensuring anonymity of responses, and all data were stored securely with access limited to the investigator. Ethical approval was obtained from the University of Pretoria Faculty of Health Sciences Research Ethics Committee (reference number 817/2020).

Descriptive statistics, including mean, median, standard deviation, and interquartile range, were used to summarise continuous variables. Frequencies and proportions were used to describe categorical variables. Given the small subgroup sizes and low expected cell counts, Fisher’s exact test was used to compare resource use across training levels. A p-value of <0.05 was considered statistically significant. Statistical analysis was performed using Python (version 3.12.13). One social media platform (Google+) was retained from the original questionnaire; however, given that this platform is no longer active, responses to this item were not included in the interpretation of social media use.

Free-text responses were analysed using a simple content analysis approach. Responses were reviewed by the investigators and grouped into categories based on recurring themes related to reasons for preferring or not preferring different resource types. Representative quotes were selected by the investigators to illustrate each category. This process was intended to provide contextual depth to the quantitative findings rather than to perform formal qualitative analysis.

All eligible EM registrars and specialists affiliated with the two universities were invited to participate. At the time of the study, this comprised a total of 66 individuals. No formal sample size calculation was performed, as the study aimed to capture the entire accessible population. A total of 55 responses were received, yielding a response rate of 83%, which is considered acceptable for survey-based research and supports the representativeness of the findings within this context.

## Results

A total of 55 responses were received. The population demographics and characteristics are described in Table 1 below. Of the respondents, the majority (65.5%) were female. Respondents between the ages of 25 and 35 accounted for 70.9% of those surveyed. Wits University accounted for 67.3% of respondents while UP accounted for 32.7%. Twenty-three-point-six percent of respondents were specialists while 76,4% were registrars.

**Table 1:** Population demographics and characteristics.

Variable	N (%)
Gender	
Male Female Other	19(34.5) 36(65,5) 0(0)
Age	
25–35 35–45 45–55	39(70.9) 11(20.0) 5(9.1)
University	
Wits UP	37(67,3) 18(32,7)
Highest level of EM education completed	
Specialists Registrars Year 1 Year 2 Year 3 Year 4	13(23,6) 42(76,4) 21(38,2) 5(9,1) 5(9,1) 11(20,0)
Devices used to access FOAM	
Smartphone Computer/ laptop Tablet device	55(100) 41(74,5) 25(45,5)
Form of connectivity used most often	
Personal Wifi Network data University Wifi Cannot access due to lack of connectivity	29(52.8) 23(41.8) 3(5,5) 0(0)
Impact of FOAM resources on clinical practice: Specialists	
Very much Somewhat Not at all	10(83.3) 2(16.7) 0(0)
Impact of FOAM resources on clinical practice: Registrars	
Very much Somewhat Not at all	33(76.7) 10(23.3) 0(0)
How often are references for Social media/FOAM resources checked: Specialists	
Always 75% of the time 50% of the time 25% of the time Never	2(16.7) 2(16,7) 3(25.0) 5(41.6) 0(0)
How often are references for Social media/FOAM resources checked: Registrars	
Always 75% of the time 50% of the time 25% of the time Never	3(7.1) 5(11.9) 9(21.4) 13(31,0) 12(28.6)

*EM = Emergency Medicine; FOAM = Free Open Access Medical Education; UP = University of Pretoria; Wits = University of the Witwatersrand; WiFi = wireless internet connectivity.*

*Registrar = postgraduate specialist trainee (equivalent to a resident in other settings); Consultant = qualified emergency medicine specialist*.

All of the respondents reported using smartphones to access modern educational resources. In addition, computers were used to access FOAM by 74.5% of respondents while tablets were used by 45,5%. Personal Wifi (52.7%) and network data (41.8%) were most often used to access these resources. University Wifi was only used by 5.5% of respondents. None of the respondents reported being unable to access FOAM resources due to lack of connectivity.

Of the respondents in the study, the majority (78.2%) reported that FOAM resources impacted their clinical practice very much. Only 7.3% of the respondents reported always checking the references for social media/FOAM resources while 22.0% reported never checking them.

**Fig. 1:**
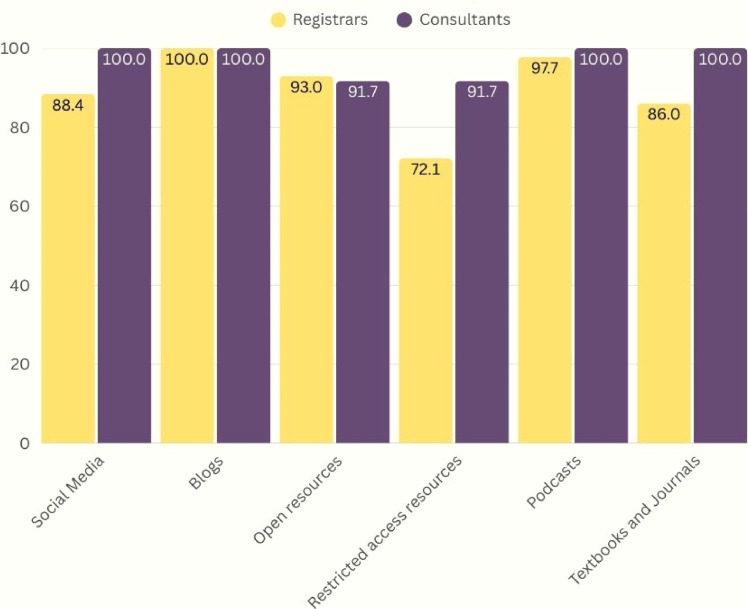
Usage of medical education resources among Emergency Medicine registrars and specialists in Gauteng.

Among the EM faculty in Gauteng, blogs are the most common modern educational resource used, with 100% of respondents reporting its use. Restricted access resources are reported to be used by 72.1% of registrars and 91.7% of specialists. Traditional resource use in the form of textbooks and journals were used by 86% of the registrars surveyed while all specialists reported use of textbooks and journals (Fig. 1).

When comparing the utilisation of different resource types across the various years of study, no statistically significant differences were identified (Table 2).

**Table 2:** Patterns of resource use across training levels.

	Consultant n (%)	1st year n (%)	2nd year n (%)	3rd year n (%)	4th year n (%)	Total n (%)	P-Value
Social media	13(100)	17(81)	5(100)	5(100)	10(91)	50(91)	0.37
Blogs	13(100)	21(100)	5(100)	5(100)	11(100)	55(100)	1.00
Open access resources	12(92)	18(86)	5(100)	5(100)	11(100)	51(93)	0.85
Restricted access resources	11(85)	14(67)	3(60)	5(100)	9(82)	42(76)	0.46
Podcasts	12(92)	21(100)	5(100)	5(100)	11(100)	54(98)	0.62
Textbooks and journals	13(100)	17(81)	5(100)	5(100)	9(82)	49(89)	0.37

The majority of respondents perceive the quality of social media, open resources, textbooks, and journals, to be acceptable. Among the respondents, 52.7% rated the quality of blogs as an educational resource as excellent, while 50.9% held the same perception for podcasts. Of the 55 respondents, one respondent rated the quality of social media and textbooks as poor, whereas 2 respondents perceived restricted access resources, podcasts, and journals to be of poor quality.

When asked about their perception of how up to date educational resources are, 47.3% of respondents rated textbooks as poor in this regard. In contrast, 49.0% of respondents perceived blogs and podcasts as excellent in terms of currency.

The reasons reported for the use of FOAM and social media resources among our study were most often for emergency medicine education (92.7%). This was followed by procedural skills training (76.4%) and diagnostic/imaging interpretation (74.5%). Investigating controversial topics in EM accounted for 72.7% while point-of-care learning was the least reported reason for using FOAM resources at 69.1%.

Table 3 illustrates the frequency with which selected educational resources are used among emergency medicine practitioners in our study population. The table presents the most frequently used resources group by type of resource.

**Table 3:** Frequency of use of selected resources in Emergency Medicine education (%).

	Daily	Weekly	Monthly	Once or twice ever	Never
Social media					
X (Twitter) Facebook Instagram	34.9 14.3 22.7	27.9 30.6 22.7	2.3 4.1 9.1	9.3 18.4 9.1	25.6 32.7 36.4
Blogs					
LITFL R.E.B.E.L EM EMCrit EMCases	32.7 20.8 20.8 20.8	50.9 47.2 37.7 50.9	16.4 26.4 26.4 13.2	0.0 5.7 9.4 5.7	0.0 0.0 1.9 1.9
Open Access					
Medscape WikiEM	11.8 4.5	45.1 36.4	13.7 25.0	19.6 9.1	9.8 25.0
Restricted access					
UpToDate University library	19.6 2.6	39.1 30.8	19.6 12.8	17.4 15.4	4.3 38.5
Podcasts					
EMCrit Project EMCases CRACKcast	36.5 28.3 14.5	34.6 41.5 23.6	19.2 20.8 10.9	5.7 9.7 21.8	3.8 0 12.7
Textbooks and Journals					
Printed textbooks Electronic textbooks Printed journals Electronic journals	12.5 23.8 0.0 15.6	10.0 28.6 11.1 44.4	27.5 16.7 8.3 35.6	20.0 28.6 22.2 4.4	30.0 2.4 58.3 0.0

FOAM = Free Open Access Medical Education; LITFL = Life in the Fast Lane; EM = Emergency Medicine.

Percentages are calculated based on available responses; totals may not equal 100% due to non-response for specific items.

The EMCrit podcast was the most frequently used resource overall, with 36.5% of respondents reporting daily use, 34.6% weekly use, and 19.2% monthly use. Similarly, the Life in the Fast Lane (LITFL) blog was highly utilised, with 32.7% using it daily, 50.9% weekly, and 16.4% monthly. The EM Cases podcast also demonstrated significant engagement, with 28.3% reporting daily use, 41.5% weekly use, and 21.8% monthly use. Among social media platforms, X (formerly Twitter) were reported as the most frequently used for educational purposes, with the highest rates of daily engagement among respondents.

Use of electronic textbooks and journals was less frequent but still notable. Electronic textbooks were used daily by 23.8%, weekly by 28.6%, and monthly by 16.7% of respondents. Electronic journals saw 15.6% daily use, 44.4% weekly use, and 35.6% monthly use. Restricted access platforms such as ENLIGHTENme, Challenger, and CME resources were the least utilised. No respondents reported daily use of these platforms. Notably, 94.6% reported never using Challenger, and 91.9% never used ENLIGHTENme. University Library use is low among our respondents, with only 1 registrar reporting daily use. Among specialist, 5 out of 13 report weekly use, while among registrars 7 out of 42 report weekly use and 9 report never using this resource.

Table 4 depicts the reasons given by our respondents for preferring or not preferring resource use. The majority of respondents cited that modern resources were easy to use, readily available, more succinct and were perceived to be more current than traditional resources. Textbooks were reported to be preferred due to more rigorous peer-review process and were seen as better for a foundation of knowledge, while they are perceived to be more outdated than modern resources. Journal articles were reported to be preferred for more in-depth review of topics, but were perceived to be tedious and requiring critical appraisal before the information can be used.

**Table 4:** Reasons for preferring/ not preferring resource use.

**Modern Resources: Prefer use**

*“Prefer to use. Easily available, new concepts and news quicker out there.”*
*“I prefer to, because most of them have summarised and up to date information unlike books.”*
*“Busy work life, therefore easier to access podcasts when driving. However, information more easily retained with reading. Textbooks often have laboured paragraphs. Online resources are straight to the salient points.”*
*“I prefer more modern sources and they are more accessible, current and discuss the controversies more often.”*
*“Prefer to use them as they are readily available, easy to access at the bedside.”*
*“I prefer modern educational resources as they are updated more regularly and more up to date and there’s a lot of good quality FOAM Ed.”*

**Modern Resources: Do not prefer use**

*“I do use the educational resources mentioned above but with discretion. There is a rating for blogs and podcast linked to their quality and how current their information/content is. I believe everyone who refers to these resources should know this rating”*

**Traditional Resources: Prefer use**

*“Prefer to use textbook. Information made easy to understand.”*
*“I use textbooks as a foundation for a more in-depth understanding. I do not prefer one above the other. They're not interchangable.”*
*“I use the textbooks to aid in studying basic principles but not for current or acutely changing practices”*
*“I prefer to use textbooks as they go through more rigorous peer-review and editing processes.”*
*“Prefer to use journals for rarer entities, deeper explanation of topics and themes.”*
*“I prefer to use textbooks, journals, as it provides an in-depth review of a single topic”*

**Traditional Resources: Do not prefer use**

*“I find textbooks often have outdated management strategies and while they are still a great resource particularly in terms of understanding presentation and pathophysiology they can quickly become less relevant in terms of management as guidelines change.”*
*“Journals and research papers require extra effort of appraisal before they can be used to learn from or change practice. They are an important source of learning but are tedious for everyday use.”*
*“I find journals difficult to follow. I often get bored when reading through large volumes of text.”*
*“Expensive and often outdated however they provide a good backbone for studying”*
*“I do not prefer to use textbooks as mostly outdated protocols and difficult to access at work.”*
*“I prefer not to use textbooks. I find them cumbersome to carry around. E-books are manageable but I prefer blogposts as they are already summarized and I feel like they are more current”*

*FOAM = Free Open Access Medical Education; EM = Emergency Medicine.*

*Responses are representative excerpts from free-text responses and have been edited minimally for clarity without altering meaning*.

## Discussion

This study describes the use of various modern and traditional educational resources in the EM faculty of Gauteng. Modern resources include social media and FOAM (e.g., podcasts, blogs, videocasts), while traditional resources refer to textbooks as well as electronic and printed journal articles. Our findings show high usage of FOAM resources, but traditional resources still maintain an important role, particularly for foundational knowledge and in-depth reviews.

Kleynhans et al. [5] conducted a similar study in the Western Cape, providing a useful comparison. While their study population preferred traditional resources overall, our respondents indicated a preference for both modern and traditional formats, with the exception of printed journals. In contrast to Kleynhans et al. [5], we found 100% of specialists in Gauteng use social media for medical education (vs. 81.5% in Cape Town), while registrar use was lower (88.1%vs. 91.2%). The lower reported use of social media among registrars compared to specialists in our study may reflect a preference for more structured resources in the earlier stages of training, or a lack of awareness of the educational value embedded within social platforms. Blog use in our population was 100%, compared to 74.1% of specialists and 82.4% of registrars in the Cape Town cohort. This may reflect growing trust in FOAM tools and improved quality controls such as the AIR score [6]. Restricted access resources (e.g., EM:RAP, ENLIGHTENme) were the least used in both studies. However, 72.1% of our respondents reported using them compared to 55.9% in the Cape Town study [5], suggesting increased engagement with these platforms in our setting.

Among the various FOAM resources available, the EMCrit podcast emerged as the most frequently used individual resource in our study, with 36.5% of respondents using it daily, 34.6% weekly, and 19.2% monthly. However, EMCrit transitioned to a partially restricted-access model from approximately 2023, overlapping with our data collection period. As such, our findings likely reflect a transitional phase in access, with users potentially engaging with both legacy open-access content and newer restricted components. This may suggest that high-quality FOAM resources may retain popularity despite evolving access models. Life in the Fast Lane (LITFL) was also highly utilised, with 32.2% of respondents reporting daily use, 50.9% weekly, and 16.4% monthly. LITFL’s popularity in our cohort is consistent with findings from Botswana, where 100% of EM trainees used it, although usage was lower in Papua New Guinea [7]. Its broad appeal may stem from its comprehensive, up-to-date content and user-friendly format, making it an accessible tool for rapid learning and clinical decision support.

The EMCases podcast followed closely in popularity, with 28.3% using it daily, 41.5% weekly, and 21.8% monthly, indicating that audio-based FOAM content is a key component of educational engagement, particularly during commutes or off-shift hours. The strong preference for podcast-based FOAM resources observed in our study is echoed in a recent electronic survey conducted among EM trainees at four Southern African universities, which explored podcast format and content preferences (Ekambaram et al. [7]). The study highlights that trainees value podcasts that are concise, case-based, and clinically relevant — characteristics that align with the popularity of platforms such as EMCrit and EM Cases in our population. When it comes to social media platforms, X (formerly Twitter) was the most frequently used for EM education on a daily basis. This likely reflects the dynamic, real-time nature of these platforms, allowing practitioners to stay up to date with emerging literature, clinical controversies, and expert discussions.

Use of university library resources was notably low among our respondents, with only one registrar reporting daily use and a substantial proportion of registrars (21.4%) indicating they never accessed these resources. This may reflect barriers such as limited off-site access, user-unfriendly platforms, or a growing preference for more accessible and immediately applicable FOAM resources. Among specialists, university library use is higher, likely due to them being more aware of this platform. The high frequency of use of open-access FOAM resources such as EMCrit, LITFL, and EM Cases, compared to the minimal use of restricted-access platforms like ENLIGHTENme and Challenger, suggests a clear preference for freely available, high-yield content—likely driven by ease of access, cost considerations, and time efficiency in a resource-constrained clinical environment.

The pattern of resource use among EM faculty in Gauteng aligns with several international contexts. In Canada, there is similarly high use of online textbooks and journals among EM trainees [8]. In Botswana and Papua New Guinea (PNG), 100% of respondents reported using traditional resources [1], mirroring the continued relevance of textbooks and journals observed in our cohort. By contrast, resource preferences in the United States differ markedly: a national survey among EM residents found that podcasts were the dominant modality (used by 70.3%), while textbook use was significantly lower at 54.3%, and journal use at just 36.5% [8]. These differences may reflect greater reliance on asynchronous, audio-based learning in high-income settings with well-established FOAM ecosystems

Kleynhans et al. [5] identified common FOAM barriers (e.g., lack of awareness, poor structure, unverified content), but our population did not report any significant barriers. Instead, FOAM resources were described as accessible, current, and concise. In contrast to previous studies conducted in other LMIC settings—such as Botswana and Papua New Guinea—where lack of reliable internet access and data affordability were identified as major barriers to FOAM use [1], all respondents in our study reported adequate connectivity. This suggests a relatively well-developed digital infrastructure in urban academic centres in Gauteng, and may reflect an improving national trend in access to online educational resources.

A notable finding is that 21.8% of respondents never check FOAM references, significantly higher than the 5.9% reported in the US (Mallin et al. [9]). This highlights an area of concern in critical appraisal skills. This presents an opportunity for targeted educational interventions. Training programmes could incorporate structured teaching on digital resource appraisal, including the use of validated FOAM evaluation frameworks (for example the AliEM AIR score [8]). In addition, integrating guided FOAM discussions, journal club-style reviews of online content, and explicit modelling of critical appraisal by faculty may help develop more discerning use of these resources. Embedding these skills within formal curricula is likely to be particularly important in settings where FOAM constitutes a major component of self-directed learning as in our setting. Although both traditional and modern resources were rated “acceptable” or “excellent,” nearly half (47.3%) rated the currency of traditional textbooks as poor, reinforcing perceptions of them as outdated.

The primary reason cited by respondents for using FOAM and social media resources was emergency medicine education, with 93% of participants identifying this as their main motivation. This aligns with international findings — such as the Canadian survey by Purdy et al. [10] — where 98% of EM trainees also used online educational resources primarily for EM learning. The high percentage in our study reinforces the role of FOAM as a core learning tool in South African postgraduate EM training. Beyond general education, procedural skills training (76%) and diagnostic/imaging interpretation (75%) were also frequently cited. Interestingly, point-of-care learning (69%), though still commonly reported, was the least cited reason for FOAM use. This is notably lower than figures from high-income countries such as Canada, where 86% of EM trainees and 82% of program directors reported using online resources to answer clinical questions in real time [10]. This may reflect differing clinical workflow structures with reliance on established protocols and senior input. Practical constraints such as time pressure, poor bedside internet connectivity or lack of computers in certain EDs may further limit the use of FOAM during patient care. In addition, clinicians may be cautious about applying FOAM content directly to patient care without formal verification or contextualisation. Overall, the reasons cited reflect that while FOAM is embraced as a supplementary educational tool, there remains room to strengthen its integration into formal EM training — particularly in promoting its use at the bedside and for clinical decision-making in complex scenarios.

## Study limitations

This study's limitations include the use of a cross-sectional design with census sampling and a relatively small sample size, which could introduce selection and non-responder bias. The distribution of the survey via programme heads may have introduced response bias or a perceived expectation to participate, although this was mitigated by the anonymous and voluntary nature of the survey. As data were self-reported, responses may be subject to social desirability bias, with participants potentially over-reporting engagement with educational resources. Additionally, the focus on Gauteng-based academic centers as well as the imbalance in representation between institutions, with a larger proportion of responses from one programme, may limit the generalisability of the findings and introduce institutional bias. The inclusion of Google+ as a response option represents a limitation of the adapted questionnaire, as the platform is no longer active. It is possible that respondents interpreted this as referring to Google as a search engine, introducing ambiguity in this variable. This item was therefore excluded from interpretation in the analysis. FOAM is a constantly evolving landscape, with new blogs, podcasts, and platforms emerging regularly. The popularity or accessibility of specific resources may shift over time, potentially limiting the long-term generalisability of our findings.

## Conclusion

This study highlights that among emergency medicine faculty in Gauteng, the use of modern educational resources—such as social media platforms, blogs, and podcasts—is both widespread and deeply integrated into daily practice. Our findings suggest that, while FOAM was widely used in our cohort, traditional resources such as textbooks and peer-reviewed journals continue to play an important role, particularly for foundational knowledge and in-depth learning. Although no major barriers were reported to accessing FOAM resources, suggesting growing awareness and confidence in these platforms, gaps remain in critical appraisal habits, with a significant proportion of respondents not routinely verifying sources—highlighting an area for educational reinforcement. The findings can inform the design of more balanced, accessible, and effective EM training programs. Understanding the perceived strengths and weaknesses of each resource type can guide educators in curating high-quality content and integrating FOAM strategically into formal curricula. The study also lays a foundation for further research into educational resource use across other healthcare disciplines and geographic regions.

## Dissemination of results

Results of this study will be shared to the relevant program coordinators at the Gauteng-based medical schools. Results will also be shared to the EM curriculum redesign work group for consideration during the curriculum redesign.

## Declaration of generative AI and AI-assisted technologies in the writing process

During the preparation of this work the author(s) used Grammarly in order to improve language and readability After using this tool/service, the author(s) reviewed and edited the content as needed and take(s) full responsibility for the content of the publication.

## CRediT authorship contribution statement

**Almien Smit:** Conceptualization, Methodology, Formal analysis, Investigation, Data curation, Writing – original draft. **Vidya Lalloo:** Supervision, Methodology, Writing – review & editing. All authors have reviewed the final submission and are accountable to the contents.

## Appendix Supplementary materials

Image, application 1

## References

[1] Thurtle N., Banks C., Cox M., Pain T., Furyk J. (2016). Free Open Access Medical Education resource knowledge and utilisation amongst Emergency Medicine trainees: a survey in four countries. Afr J Emerg Med.

[2] Burkholder T.W., Bellows J.W., King R.A. (2018). Free open access medical education (FOAM) in emergency medicine: the global distribution of users in 2016. West J Emerg Med.

[3] Hootsuite (2026). Social media statistics for 2026 [Internet]. https://blog.hootsuite.com/social-media-statistics/.

[4] Google (2026). https://support.google.com/analytics/answer/2731565.

[5] Kleynhans A.C., Oosthuizen A.H., van Hoving D.J. (2017). Emergency medicine educational resource use in Cape Town: modern or traditional?. Postgrad Med J.

[6] Chan T.M., Bhalerao A., Thoma B., Trueger N.S., Grock A. (2019). Thinking critically about appraising FOAM. AEM Educ Train.

[7] Ekambaram K., Lamprecht H., Lalloo V., Caruso N., Engelbrecht A., Jooste W. (2021). An electronic survey of preferred podcast format and content requirements among trainee emergency medicine specialists in four Southern African universities. Afr J Emerg Med..

[8] Chan T.M., Grock A., Paddock M., Kulasegaram K., Yarris L.M., Lin M. (2016). Examining reliability and validity of an online score (ALiEM AIR) for rating free open access medical education resources. Ann Emerg Med.

[9] Mallin M., Schlein S., Doctor S., Stroud S., Dawson M., Fix M. (2014). A survey of the current utilization of asynchronous education among emergency medicine residents in the United States. Acad Med.

[10] Purdy E., Thoma B., Bednarczyk J. (2015). The use of free online educational resources by Canadian emergency medicine residents and program directors. CJEM.

