# Supplementary material for: Traditional and modern educational resource use in Emergency Medicine registrars (trainees) and specialists in South Africa
Source: Afr J Emerg Med. 2026 Jun 22;16(3):100985. doi: 10.1016/j.afjem.2026.100985 (PMC13315808; doi:10.1016/j.afjem.2026.100985)
Supplement: Supplementary file 1 [file mmc1.docx]

APPENDIX A : STUDY QUESTIONNAIRE

Utilisation of educational resources in the Emergency

Medicine Faculty of Gauteng

Principle investigator: Dr Almien Smit

Supervisor: Prof A Engelbrecht

Institution: University of Pretoria

Daytime telephone number: 0834587733 / +27 12 354 2243

Dear Prospective Research Participant

Dear Dr

1) INTRODUCTION

You are invited to volunteer for a research study. The information in this document is provided

to help you to decide if you would like to participate. Before you agree to take part in this study,

you should fully understand what is involved. If you have any questions, which are not fully

explained in this document, do not hesitate to ask the researcher. You should not agree to take

part unless you are completely happy with the kind of questions that will be asked.

2) THE NATURE AND PURPOSE OF THIS STUDY

There is a major international trend away from conventional teaching and learning, which

used to be mainly handbook- and journal-based, towards integrating online resources and

social media platforms into daily emergency medical practice and continued education. It is

therefore important to determine which educational resources Emergency Medicine faculty

in Gauteng are using before one can extrapolate how the use of online resources and

social media may affect the practice and outcomes of healthcare.

3) EXPLANATION OF PROCEDURES AND WHAT WILL BE EXPECTED FROM

PARTICIPANTS

This study involves answering some questions regarding the educational resources that you use.

We would like you to complete a questionnaire. It will take approximately 10 minutes. The survey

is on a survey monkey platform and results will be accessible to the investigators only. We will

not be available to help you with the questionnaire. Please do not type your name on the

questionnaire. This will ensure that your answers are kept confidential (so nobody will know

what you have answered).

The questionnaire consists of Two parts:

Part 1: Socio-demographic information which involves answering some questions about your age,

gender, year of study, etc.

Part 2: Questionnaire on educational resources you use, including social media, FOAM , and

traditional resources. We also ask how you use them, why you prefer a certain type of resource

and how it impacts on your practice.

4) RISK AND DISCOMFORT INVOLVED

There is no foreseeable physical discomfort or risk involved.

.

5) POSSIBLE BENEFITS OF THIS STUDY

The knowledge gained from this study would help the faculty of Emergency Medicine in

Gauteng to evaluate their current teaching strategies and adapt it if needed, leading to

improved faculty development and ensuring that students and lectures stay on par with

these rapidly evolving and possibly powerful resources.

6) ETHICS APPROVAL

This Protocol was submitted to the Faculty of Health Sciences Research Ethics Committee,

University of Pretoria, Medical Campus, Tswelopele Building, Level 4-59, Telephone numbers

012 356 3084 / 012 356 3085 and written approval has been granted by that committee. The

study has been structured in accordance with the Declaration of Helsinki (last update: October

2013), which deals with the recommendations guiding doctors in biomedical research involving

humans. A copy of the Declaration may be obtained from the investigator should you wish to

review it.

7) INFORMATION

If you have any questions concerning this study, you may contact: Dr Almien Smit at 0834587733

or almien.boshoff@gmail.com

8) CONFIDENTIALITY

All records from this study will be regarded as confidential. All results will be published or

presented in such a way that it is not possible to identify the participants.

9) COMPENSATION

You will not be paid to take part in the study. There are no costs involved for you to be part of

the study.

9) CONSENT TO PARTICIPATE IN THIS STUDY

 I confirm that the person requesting my consent to take part in this study has told me

about the nature and process, any risks or discomforts, and the benefits of the study.

 I have also received, read and understood the above written information about the study.

 I have had adequate time to ask questions and I have no objections to participate in this

study.

 I am aware that the information obtained in the study, including personal details, will be

anonymously processed and presented in the reporting of results.

 I understand that I will not be penalised in any way should I wish to discontinue with the

study and my withdrawal will not affect my employment or student status.

 I am participating willingly.

Questionnaire: Modern vs Traditional resource use in Emergency Medicine Gauteng

1. By continuing with this survey, you confirm that you have read and understood

the disclaimer in the invitation email and that you voluntarily agree to participate.

Answer Options

Start the survey

I do not want to take part in this survey.

Please remind me to complete the survey at a later stage

2. Age

Please specify

3. Gender

Answer Options

Male

Female

Other (please specify): __________________________________

4. Profession

Answer Options

Emergency medicine registrar

Emergency medicine consultant

5. University

Answer options

UP

WITS

6. Years completed of current degree

Answer Options

Completed

1

2

3

4

7. Do you use SOCIAL MEDIA as an educational resource? (eg Facebook, Twitter,

Instagram)

Answer Options

Yes

No

8. Please indicate how often you make use of the following SOCIAL MEDIA

platforms for educational purposes

Answer Options Weekly Monthly Once or Twice ever Never

Facebook

Twitter

Instagram

LinkedIn

Google+

Other (please specify)

9. Please indicate your perception of the quality and of how current the educational

information available on SOCIAL MEDIA is

Answer Options Poor Acceptable Excellent

Quality?

How Current?

10.

11. Do you use BLOGS as an educational resource? (eg LITFL, EMCRIT project,

Core EM)

Answer Options

Yes

No

12. Please indicate how often you make use of the following popular BLOGS for

educational purposes

Answer Options Weekly Monthly Once or Twice

Never

LITFL (Life in the Fast Lane)

ALiEM (Academic Life in Emergency Medicine)

R.E.B.E.L EM

emDocs

CanadiEM

EMBasic

EMCrit

St Emlyns

Core EM

EMCases

Don’t Forget the Bubbles

Other

Other (please specify)

13. Please indicate your perception of the quality and of how current the educational

information available on BLOGS are

Answer Options Poor Acceptable Excellent

Quality?

How Current?

14.

15. Do you use Open Educational Resources? (websites eg.medscape, wikiEM

ACEP now)

Answer Options

Yes

No

16. Please indicate how often you make use of the following popular OPEN

EDUCATIONAL RESOURCES

Answer Options Weekly Monthly Once or Twice

Never

WikiEM

GMEP (Global Medical Education Project)

Medscape

EMedicine

ACEP now

Other

17. Please indicate your perception of the quality and of how current information

available in OPEN EDUCATIONAL RESOURCES are

Answer Options Poor Acceptable Excellent

Quality?

How Current?

18.

19. Do you use RESTRICTED ACCESS EDUCATIONAL RESOURCES?

(egUpToDate)

Answer Options

Yes

No

20. Please indicate how often you make use of the following RESTRICTED ACCESS

EDUCATIONAL RESOURCES

Answer Options Weekly Monthly Once or Twice

Never UpToDate

ENLIGHTENme

Challenger

CME

University-specific (eg. Library)

Other

Other (please specify)

21. Please indicate your perception of the quality and of how current RESTRICTED

ACCESS EDUCATIONAL RESOURCES are

Answer Options Poor Acceptable Excellent

Quality?

How Current?

22. What types of RESTRICTED ACCESS EDUCATIONAL RESOURCES do you

currently use?

Answer Options

Free (need to subscribe / log-in only)

Paid, own account

Paid, by employer or academic institute

Paid, shared group subscription

23.

24. Do you listen to podcasts as an educational resource?

Answer Options

Yes

No

25. Please indicate how often you make use of PODCASTS

Answer Options Weekly Monthly Once or Twice

Never

EMcases

EMCRIT podcast

EM:RAP

Annals of Emergency medicine

ALiEM podcast

ERCast

Core EM

EM Basic

CRACKCast

AAEM

Emergency Medical minute

Ultrasound Podcast

Other

Other (Specify)

26. Please indicate your perception of the quality and of how current PODCASTS are

Answer Options Poor Acceptable Excellent

Quality?

How Current?

27.

28. Do you use Textbooks and Academic Journals?

Answer Options

Yes

No

29. Please indicate how often you make use of TEXTBOOKS and ACADEMIC

JOURNALS

Answer Options Weekly Monthly Once or Twice

Never

Textbooks, printed

Textbooks, electronic

Journals, printed

Journals, electronic

Other

Other (please specify)

30. Please indicate your perception of the quality and of how current information

available in TEXTBOOKS are

Answer Options Poor Acceptable Excellent

Quality?

How Current?

31. Please indicate your perception of the quality and of how current information

available in ACADEMIC JOURNALS are

Answer Options Poor Acceptable Excellent

Quality?

How Current?

32. Please briefly comment if you prefer to/ prefer not to use Modern educational

resources such as blogs, podcasts, social media etc and state your reason

why/why not

33. Please briefly comment if you prefer to/ prefer not to use textbooks and journals

and state your reason why/why not

34. What is the purpose for which you access FOAM and social media resources?

Answer options

EM education

procedural skills training

diagnostic/imaging interpretation

answering questions at the point of patient care

investigating controversial topics in EM

I don’t use FOAM/social media

Other (please specify)

35. What devices do you use to access FOAM and social media resources

Answer Options

Computer / Laptop

Tablet device

Smart Phone

Other (please specify)

36. What form of connectivity do you use most often to access these resources?

Answer options

Network data

personal wifi

University wifi

unable to access due to lack of data

37. How often do you check the references of the Social media/FOAM resources that

you use?

Answer options

Always

75% of the time

50% of the time

25% of the time

I don’t use FOAM/social media

Never

38. How has FOAM and social media resources impacted your clinical practice?

Answer options

Very much

Somewhat

Not at all
